# Biofilms in Periprosthetic Orthopedic Infections Seen through the Eyes of Neutrophils: How Can We Help Neutrophils?

**DOI:** 10.3390/ijms242316669

**Published:** 2023-11-23

**Authors:** Carla Renata Arciola, Stefano Ravaioli, Rasoul Mirzaei, Paolo Dolzani, Lucio Montanaro, Maria Daglia, Davide Campoccia

**Affiliations:** 1 Laboratory of Immunorheumatology and Tissue Regeneration, Laboratory of Pathology of Implant Infections, IRCCS Istituto Ortopedico Rizzoli, Via di Barbiano 1/10, 40136 Bologna, Italy; lucio.montanaro@unibo.it; 2 Department of Medical and Surgical Sciences (DIMEC), University of Bologna, Via San Giacomo 14, 40126 Bologna, Italy; 3 Laboratorio di Patologia delle Infezioni Associate all’Impianto, IRCCS Istituto Ortopedico Rizzoli, Via di Barbiano 1/10, 40136 Bologna, Italy; stefano.ravaioli@ior.it (S.R.); davide.campoccia@ior.it (D.C.); 4 Venom and Biotherapeutics Molecules Laboratory, Medical Biotechnology Department, Biotechnology Research Center, Pasteur Institute of Iran, Tehran 1316943551, Iran; rasul.micro92@gmail.com; 5 Laboratorio di Immunoreumatologia e Rigenerazione Tissutale, IRCCS Istituto Ortopedico Rizzoli, Via di Barbiano 1/10, 40136 Bologna, Italy; paolo.dolzani@ior.it; 6 Department of Pharmacy, University of Napoli Federico II, Via D. Montesano 49, 80131 Naples, Italy; maria.daglia@unina.it

**Keywords:** neutrophils, implant infections, periprosthetic infections, orthopedic implants, implant failure, bacterial biofilms

## Abstract

Despite advancements in our knowledge of neutrophil responses to planktonic bacteria during acute inflammation, much remains to be elucidated on how neutrophils deal with bacterial biofilms in implant infections. Further complexity transpires from the emerging findings on the role that biomaterials play in conditioning bacterial adhesion, the variety of biofilm matrices, and the insidious measures that biofilm bacteria devise against neutrophils. Thus, grasping the entirety of neutrophil–biofilm interactions occurring in periprosthetic tissues is a difficult goal. The bactericidal weapons of neutrophils consist of the following: ready-to-use antibacterial proteins and enzymes stored in granules; NADPH oxidase-derived reactive oxygen species (ROS); and net-like structures of DNA, histones, and granule proteins, which neutrophils extrude to extracellularly trap pathogens (the so-called NETs: an allusive acronym for “neutrophil extracellular traps”). Neutrophils are bactericidal (and therefore defensive) cells endowed with a rich offensive armamentarium through which, if frustrated in their attempts to engulf and phagocytose biofilms, they can trigger the destruction of periprosthetic bone. This study speculates on how neutrophils interact with biofilms in the dramatic scenario of implant infections, also considering the implications of this interaction in view of the design of new therapeutic strategies and functionalized biomaterials, to help neutrophils in their arduous task of managing biofilms.

## 1. Introduction

Implant infection still represents a serious complication of orthopedic prosthetic surgery [1,2]. Indeed, this peculiar post-surgical infection displays an aptitude for persisting, a reluctance to be eradicated, and an inclination to cause implant failure [3,4,5]. In most cases, the presence of a biofilm characterizes implant infections.

Bill Costerton, the father of biofilm, first introduced the notion of “biofilm” to describe a community of bacteria adhering to river rocks [6]. Bacteria form biofilms where nutrients are lacking, or in other disadvantageous conditions. Indeed, by joining together in a consortium, they can cope with unfavorable conditions [7,8,9,10,11]. Costerton’s pioneering studies indicated that bacteria pursue the biofilm lifestyle in various natural environments. In the human body, biofilm helps bacteria to survive, it protects them against the host’s defenses, and it strengthens their pathogenetic potential [11,12,13,14,15,16]. Thus, in various pathological contexts, such as that of implant infections, bacterial biofilms appear to be the main cause of infection persistence, aggressiveness, and destructiveness [17,18,19,20]. The etiological agents of orthopedic implant infections are mainly Gram-positive species belonging to the genus *Staphylococcus*, in particular *Staphylococcus aureus* and *Staphylococcus epidermidis* [19,20,21]. In other words, these are opportunistic bacteria that most frequently behave as saprophytes or mild pathogens, but which become serious pathogens in periprosthetic tissues. Indeed, they find in the *locus minoris resistentiae* of periprosthetic tissues the opportunity to thrive, adhering to the prosthesis surfaces, colonizing them, and forming biofilms. Furthermore, a loss of bone substance occurs in these infections, resulting in septic loosening of the implant. These events create the need to remove the prosthesis, together with the infected housing tissues, and then to insert a new prosthesis. These surgical procedures lead to clinical results that are not always satisfactory. In fact, in addition to partly involving demolitive surgery to remove infected tissues, re-implants are burdened by a high risk of reinfection. The outlook is that of a complex surgical therapy followed by prolonged rehabilitation treatments, which involve high socio-economic costs [1,20,21,22,23,24]. As an increasing number of prostheses are implanted every year in the world, it is very important to explore the pathogenetic mechanisms that produce these dramatic negative effects and to understand how neutrophils, our first line of defense, act in this scenario. In the implant infection site, neutrophils find not only bacteria but also biomaterials. This study scrutinizes the weapons of neutrophils and explores the multiplicity of the interactions of neutrophils with biomaterials and biofilms, while also considering strategies that could support the defensive performances of neutrophils.

## 2. Neutrophils: War Machines against Bacteria

Accounting for 50% to 70% of circulating human leukocytes, polymorphonuclear neutrophils (PMNs) are the most numerous leukocytes in the blood. They are warrior cells, and their main enemies are bacteria. Neutrophils sight bacteria that have exceeded the physical barriers of the human body. When neutrophils detect the presence of bacteria in the tissues, they flock to the site of infection in large numbers within minutes. Neutrophil migration from blood toward invading bacteria is guided by a series of chemoattractant molecules of both bacterial and endogenous origin (e.g., bacteria-derived lipids, bacteria-derived *N*-formylated peptides (fMLP or *N*-formyl-met-leu-phe), complement components (e.g., C5a), chemokines and cytokines (CXCL8, IFN-γ), and leukotrienes (LTB4)) [25,26]. Neutrophils sense chemoattractant molecules and they move towards higher concentrations, following the gradient. This process is known as *chemotaxis* or *guided cell migration* [27]. From a mechanistic point of view, the chemotactic recruitment of neutrophils to sites of infection depends on their expression of chemoattractant receptors. The formyl peptide receptor (FPR1) was the first receptor to be cloned and characterized. FPR1 exhibits high affinity for bacterial-derived peptides that contain a formylated methionine in the *N*-terminus. Dorward et al. presented a review on the role of formyl peptide receptors in governing neutrophil function [28].

**Figure 1:**
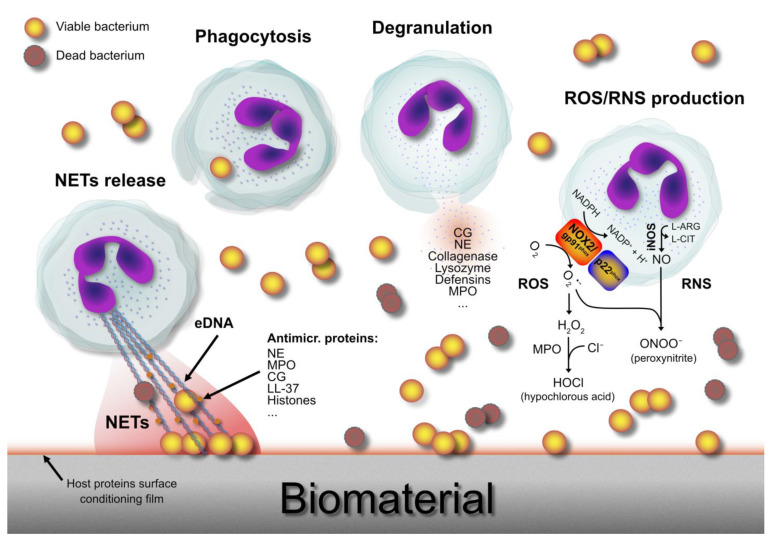
The different antibacterial tasks accomplished by neutrophils to contrast invading opportunistic pathogens at the implant–tissue interface: (1) release of antimicrobial peptides and enzymes via degranulation; (2) production of ROS and RNS; (3) release of NETs; (4) phagocytosis. Moreover, neutrophils synthesize pro-inflammatory mediators to recruit other leukocytes, thus amplifying and intensifying their antibacterial action. Legend: NE, neutrophil elastase; CG, cathepsin G; MPO, myeloperoxidase; NOX2/gp91^phox^, catalytic subunit of nicotinamide adenine dinucleotide phosphate (NADPH) oxidase (NOX) expressed in phagocytic cells; p22^phox^, a critical component of the membrane-bound superoxide-generating NOX expressed in neutrophils; iNOS, inducible nitric oxide synthase; L-ARG, L-arginine; L-CIT, L-citrulline; ROS, reactive oxygen species; RNS, reactive nitrogen species; NETs, neutrophil extracellular traps.

To reach bacteria, neutrophils must migrate through the wall of the vessel. They adhere at first loosely, then with increased avidity to the receptors exposed at different times on the surface of the endothelial cells. Namely, neutrophils are first tethered by selectins expressed on the surface of endothelial cells (E-selectin and P-selectin) and on neutrophils (L-selectin). Selectins interact with sialylated carbohydrate groups present on the surface proteins of the cellular counterpart. This results in the so-called *rolling* of neutrophils along the endothelium. Rolling, neutrophils proceed slowly, as they are tethered through selectin interactions. This allows them to recognize chemokines on the surface of the endothelium, which activates integrins. Then, ICAM-1 and VCAM-1 cell adhesion molecules turn out to be overexpressed on the endothelial cells. Thus, low-affinity selectin interactions are gradually replaced by high-affinity interactions between integrins and the cellular adhesion molecules on the endothelial cells. Once neutrophils have anchored themselves to the endothelium, they emit a contractile pseudopod, then they insinuate it through the intracellular openings of the permeabilized endothelium and, finally, thanks to the traction movement of the pseudopod, they drag themselves out of the vessel (*diapedesis*). A transcellular exit from the vessel has also been described (Filippi M.D. has recently reviewed the mechanisms of neutrophil transendothelial migration [29]). After extravasation, the life of neutrophils is short and intense, mainly aimed at the destruction of bacteria. Figure 1 illustrates the various mechanisms implicated.

As would be expected for a short-lived warrior cell, the equipment in the cytoplasmic organelles of neutrophils is small, the cytoplasm being primarily an arsenal of weapons stored in granules. Neutrophils possess a peculiar multi-lobed nucleus, very few mitochondria, a small Golgi apparatus, and a multitude of cytoplasmic granules. Each neutrophil contains about two thousand granules of at least three types: primary/azurophilic granules, secondary/specific granules, and tertiary/gelatinase granules. Sometimes a fourth type, the so-called “secretory vesicles”, is also included.

Different granule proteins and enzymatic proteins are synthesized during different stages of neutrophil differentiation. Azurophilic granules, or “primary granules”, are the first to form during cell maturation (that is why they are called primary). Azurophilic granules contain myeloperoxidases (MPO), bactericidal/permeability-increasing protein (BPI), neutrophil elastase (NE), cathepsin G (CG), and defensins [30]. Neutrophils are capable of engulfing micro-particles no larger than 10 μm, namely, micro-particles that are no larger than they are. If the particle is larger than 10 μm, neutrophils will only be able to engulf it if they can break it down through their enzymes. In a recent article, Kavanaugh et al. demonstrated that cathepsin G, released by neutrophils through degranulation, degrades S. aureus biofilms into fragments small enough to be phagocytized [31]. Specific or secondary granules are the first consumed during the acute inflammatory response. They contain alkaline phosphatase, NADPH oxidase, collagenase, histaminase, cathelicidin, lysozyme, and the iron-binding protein lactoferrin. Tertiary or gelatinase granules contain matrix metalloproteinases, such as gelatinase and collagenase. Neutrophil granules release antibacterial proteins and enzymes, either into phagosomes or into the extracellular milieu, in order to act either on phagocytized bacteria or on bacteria present in the extracellular space. Indeed, among the enzymes of granules, some work at an acidic pH and therefore they act in the acidic internal environment of phagolysosome. Enzymes working at a neutral pH, like collagenase, are extracellularly active and useful for neutrophils to open passages through the tissues.

To destroy engulfed bacteria, besides using antibacterial proteins and enzymes, neutrophils also exploit an oxidative strategy through the production of ROS. This is the so-called respiratory burst (better defined as an oxidative burst). NADPH oxidase, a multi-subunit enzymatic complex at the phagosome membrane, triggers the oxidative burst by generating superoxide anions (O_2_^•−^). In the phagosome, either spontaneously or in a reaction catalyzed by superoxide dismutase (SOD), superoxide anions combine with hydrogen ions to form hydrogen peroxide (H_2_O_2_), from which, in turn, hydroxyl radicals (HO^•^) can be formed through the Fenton reaction. ROS bactericidal action is amplified by the interaction with nitric oxide (NO). For example, superoxide anions, by reacting with nitric oxide, form peroxynitrite anions [32,33,34]. ROS can be transformed into other bactericidal products, as in the case of the hypochlorous acid (HOCl). This is formed through myeloperoxidase (MPO)-catalyzed peroxidation of chloride ions (Cl^−^) using H_2_O_2_ [31]. Hypochlorous acid oxidizes proteins, thus producing advanced oxidation protein products (AOPPs). AOPPs are regarded as biomarkers of the oxidative damage generated by NADPH oxidase in many inflammatory disease processes [35]. Hence, the research should be focused on substances or phytocomplexes capable of inducing adaptations of the antioxidant first line defense system (especially SOD) [36,37,38,39,40].

Neutrophils are equipped with numerous “pattern recognition receptors” (PRRs) to recognize evolutionarily conserved surface molecules of pathogens, the so-called pathogen-associated molecular patterns (PAMPs) [41,42,43]. Interestingly, while phagocytosis of planktonic bacteria requires “opsonization” [44,45], this does not appear to be a pre-requisite for biofilm recognition [46,47].

Among the responses of neutrophils against pathogens is the release of neutrophil extracellular traps (NETs) [48,49]. These structures are composed of deoxyribonucleic acid and antimicrobial proteins such as neutrophil elastase, myeloperoxidase (MPO), antimicrobial peptides, cathepsin G, the cathelicidin-derived antimicrobial peptide LL-37, and other proteins from neutrophil granules. The formation of extracellular traps is considered an effective strategy to immobilize pathogens too large or aggregated to be effectively killed by phagocytosis, thus preventing them from spreading. Once the bacteria have been trapped, NETs can also be successful in facilitating phagocytosis.

Importantly, activated neutrophils synthesize pro-inflammatory mediators such as cytokines and chemokines. Mediators are synthesized and stored in intracellular vesicles. During inflammation, neutrophils release pre-synthesized mediators by exocytosis.

## 3. Neutrophils and Sterile Biomaterials

The neutrophils–biomaterial interaction depends on various factors, including the peculiarities of the tissues that will house the implant: these surrounding tissues will be the real drivers of the neutrophils’ behavior, which, in turn, will induce consequential immune responses. This can be convincingly inferred from the fact that, when implanting identical materials in different tissues, different responses are elicited [50]. When a biomaterial is implanted, the immune system reacts with an acute inflammatory response followed by resolution, or with a foreign body reaction. This develops as a chronic inflammation that leads to the formation of a fibrotic encapsulation and, over time, to tissue destruction, prosthesis degradation, and the isolation of the prosthesis from the surrounding tissues. Thus, the function of the implant is compromised, and any further harmonious interplay between the biomaterial and the host tissues will be prevented [51,52,53,54,55,56].

Biomaterial plays a crucial role in addressing neutrophils’ responses, as well as the intensity and width of the tissue damage during implantation will affect what will happen in the periprosthetic tissues following implantation. Indeed, the damage measure is related to the number and diameter of the surgically truncated vessels, the extent of the bleeding, the number of platelets released, and to several molecules such as the components of the coagulation cascade, complement proteins, pro-inflammatory cytokines, chemokines, growth factors, and extra-cellular matrix (ECM) adhesion proteins. Biomaterials with a low surface area to volume ratio often induce a sustained inflammation due to the abundant presence of neutrophils, the release of NETs, the presence of giant cells, and then fibrosis [57]. The first responder cells for sterile inflammation caused by biomaterials are neutrophils. Their primary function is the setting up of an acute inflammatory state characterized by degranulation, the secretion of chemokines, and the phagocytosis of foreign materials. These are the responses of neutrophils to bacterial infections and to chemical-induced injuries. But, also, in the acute stages of an inflammation evoked by sterile implant materials, the presence of neutrophils has been observed, and neutrophils have been shown to be involved in the degradation of implant materials. A sterile inflammatory response against the implant thus involves the recruitment and activation of neutrophils, the secretion of inflammatory mediators, and the formation of NETs around the implant. Different studies underlined the crucial role that the early neutrophils’ responses play in modulating the later intervention of macrophages (and even of lymphocytes) toward a foreign body response [58,59,60].

The proteins adsorbed onto the biomaterial surface, rather than the biomaterial surface per se, mediate the response of neutrophils. In turn, the repertoire, kinetics, and conformation of the proteins adsorbed on the implant surface depend on the chemical (hydrophobicity, charge, and functional groups) and topographic (size, shape, and texture) characteristics of the biomaterial surface. Therefore, the adsorbed proteins affect many phenomena at the material–tissue interface. Natural polymers and decellularized tissues do not induce a foreign body reaction; rather, they favorably stimulate neutrophils towards a remodeling function. On the contrary, biomaterial hydrophobicity seems to exert an intrinsic immunogenicity [61,62], as underlined by the correlation between the degree of the hydrophobicity of the material surface and the levels of expression of the pro-inflammatory cytokines IFN-γ and TNF-α by innate immunity cells. On the contrary, leukocytes adhered to hydrophilic surfaces exhibited significantly decreased production of the pro-inflammatory cytokines IL-6 and IL-8, and these hydrophilic surfaces showed significantly decreased levels of foreign body giant cells [57]. Moreover, looking at the surface topography, it has been reported that, by changing the topographical characteristics of the biomaterial surface at the nanometer scale, the adsorption of proteins on the surface can be modulated [63].

Overall, these observations highlight the key role of neutrophils in the inflammatory responses to implanted prostheses, emphasizing the importance of developing more immune-compatible implant materials and of searching for new drugs to prevent fibrotic reactions to implants [64].

## 4. Neutrophils Meet Biomaterials and Bacteria

Implant material is, per se, prone to supporting bacteria by offering them micro-niches in which they can sneak, cling to a solid surface, hide, and even enter a state of dormancy. Neutrophils are too large to access niches, while common antibiotics are rather ineffective against the dormant bacterial cells.

Depending on their specific physico-chemical nature, biomaterial surfaces can diversely adsorb the many proteins present in physiological solutions, such as ECM proteins, immunoglobulins, complement activation products, and proteins of the cascade of coagulation and of the fibrinolytic system [65,66,67]. The adsorption of proteins onto biomaterials is almost instantaneous but not instantaneously conclusive. Indeed, different proteins, adsorbing and de-adsorbing, alternate on the material surface (Vroman effect). Then, interactions with blood cells occur, in primis with PMNs. PMNs orchestrate the inflammatory response in the early days after implant surgery. They also lead to the recruitment of a second wave of cells, i.e., that of monocytes/macrophages, which will become the cellular protagonists of the later phase of tissue repair [68].

In an in vivo model of implant biofilm infection by *S. aureus*, Gries et al. (2020) examined the behavior of neutrophils by means of multiphoton technology [69]. They found significant differences in the speed and direction of neutrophils’ migration and in their position in response to infected versus sterile implants. Although the neutrophils responded to the implant biofilm by moving towards it, their migration appeared to be less lively and less focused than the migration they exhibited toward the sterile implant.

These observations are consistent with those of Ghimire et al. (2019), who suggested that the delay in neutrophil recruitment in the infected sites could allow *S. aureus* to grow and form biofilm before being caught by neutrophils [70].

## 5. Bacteria Evade Neutrophils

Besides being recognized as the most prevalent cause of implant-associated infections, *S. aureus* is a prominent human pathogen in different medical fields and a leading cause of death worldwide. *S. aureus* uses different tools to evade the host’s immune defenses [71,72,73]. It can produce various evasion molecules, for instance: the extracellular adherence protein, Eap; the chemotaxis inhibitory protein of *Staphylococcus*, CHIPS; the staphylokinase, SAK; and the staphylococcal superantigen-like protein 10, SSL10. Additionally, it also expresses pore-forming toxins and modulins, including: α-hemolysin (Hla); γ-hemolysins (HlgAB and HlgCB); bi-component leukocidins such as LukED and LukAB; and phenol soluble modulins (PSMs) [74,75,76,77,78]. Proteomic and transcriptomic approaches demonstrate that *S. aureus* in biofilm expresses evasion molecules, toxins, and PSMs at high levels, while these are expressed at lower levels when it is in planktonic phase. Some of these molecules, such as Eap, are hosted in the extracellular polymeric substance, and others are released into the environment [74]. PSMs are present in biofilms both in soluble form and assembled in amyloid structures stabilizing the biofilm [78]. *S. aureus*–host interactions and recently discovered immune evasion molecules are reviewed in Howden et al. (2023) [79]. The main anti-neutrophilic properties of *S. aureus* include the ability to induce leukolysis and to inhibit neutrophils’ diapedesis, chemotaxis, and phagocytosis.

Moreover, *S. aureus* is competent to internalize into osteoblasts, thus hiding from the eyes of leukocytes. *S. epidermidis* is incompetent to internalize into osteoblasts [80,81] and it has a much smaller repertoire to use against immune defenses [82], with its main weapon being the ability to produce biofilm. *S. aureus*, too, is a formidable biofilm former.

*S. aureus* is believed to form prevalently protein-based biofilms, while *S. epidermidis* forms mostly exopolysaccharide-based biofilms. Namely, the staphylococcal exopolysaccharide biofilm component is the poly-*N*-acetylglucosamine (PNAG) molecule, also called polysaccharide intercellular adhesin (PIA) [83,84].

Among the new emerging pathogens responsible for implant orthopedic infections, *Staphylococcus lugdunensis*, a “wolf in sheep’s clothing”, is a versatile opportunist, now recognized as a highly virulent pathogen. It cannot enter osteoblasts, but able to produce biofilms with matrices of various composition [85].

In this connection, it should be noted that the interaction of neutrophils with bacterial biofilms depends not only on the bacterium’s capability to dampen or evade immune defenses, but also on the role, activation, cooperation, and reciprocal modulation that host immune cells display [86].

*S. aureus* evades or resists innate immune defenses, especially when organized in a biofilm [86]. Although planktonic bacteria can rely on multiple mechanisms to evade being killed by neutrophils (reviewed in [87]), bacteria in biofilms are not only equipped with the same weapons of planktonic bacteria, but they are also wrapped and protected by a shield capable of absorbing many of the blows of neutrophils. Phenol-soluble modulins (PSMs) are a family of toxins responsible for the hypervirulence of highly aggressive *S. aureus* strains [88,89,90].

PSMs exhibit a multiplicity of pathogenetic mechanisms, as they lyse blood cells and favor biofilm development and dissemination. In particular, the PSM-mediated killing of human neutrophils following bacterial phagocytosis might explain the failure of neutrophils when they encounter the biofilm of hypervirulent strains of *S. aureus*.

On the other hand, in vivo investigations of the multifaceted world of immune cells suggest that there are different responses against biofilms by different immune cells and their subsets [91]. Thus, type I neutrophils (PMN-I) and M1 macrophages play an important role against *S. aureus*, while M2 macrophages are associated with biofilm persistence and sepsis. High diversity and plasticity characterize macrophages. M1 and the alternative M2 are the extremes of a dynamic changing state of activation: M1 macrophages release cytokines that inhibit the proliferation of the surrounding cells and damage the contiguous tissues, while M2 release cytokines that promote the proliferation of the adjoining cells and tissue repair [92].

## 6. Biofilm and Myeloid-Derived Suppressor Cells Recruitment

Recent findings from preclinical and clinical studies suggest that, in *S. aureus* orthopedic implant infections, interleukin-12 (IL-12) promotes the recruitment of myeloid-derived suppressor cells (MDSCs) [93]. Myeloid-derived suppressor cells (MDSCs) are a population of immature neutrophils and monocytes endowed with pronounced immunosuppressive activity, whose presence connotes advanced cancer and chronic infections [94]. As far as cancer is concerned, MDSCs seem to play multiple roles. Indeed, they suppress anti-tumor immunity and promote tumor growth, differentiation, and metastasis [95]. Early findings from preclinical and clinical studies suggest that, in *S. aureus* orthopedic implant infections, MDSCs recruited to the infected site contribute to the bacterial persistence. Interleukin-12 (IL-12) favors the recruitment of MDSCs [93]. However, there is evidence that, under various circumstances, a number of chemokines, namely CXCL1, CXCL2, and CXCL5 [96], drive MDSC recruitment to the site of infection or into the tumors. The anti-inflammatory properties of MDSCs are controlled by the release of IL-10 [95,97].

In 2015, Tebartz et al. recognized, in *S. aureus* chronic infections, an immunosuppressive condition. T-lymphocytes were not the cells responsible for this immunosuppression, unlike what might have been expected (it is known that regulatory T cells, also called suppressors, induce apoptosis in activated lymphocytes to deactivate self-directed immune responses, thus safeguarding self-tolerance and preventing autoimmunity) [98]. In *S. aureus* chronic infections, the observed immunosuppressive phenomenology was attributable to the peculiar immature cells MDSCs.

MDSCs can be distinguished into two groups: granulocytic MDSCs (G-MDSCs, also indicated as PMN-MDSCs) and monocytic MDSCs (M-MDSCs) [99]. The relationship between biofilms and MDSCs is very intriguing and is still not exhaustively clarified. In the in vivo experimental study of Heim et al., MDSCs surrounded *S. aureus* biofilm [100]. In the in vitro and in vivo studies of Peng et al. (2017) [101], MDSCs were found to be turning into macrophages when stimulated by *S. aureus* biofilms.

MDSCs observed in in vivo peri-prosthetic infections belong to the granulocytic lineage [102]. In a newly published study, Aldrich et al., by using single-cell RNA sequencing in a mouse model of *S. aureus* craniotomy infection, revealed the transcriptional heterogeneity of both resident microglial cells and infiltrating granulocyte cells. In the brain, the analysis identified the transition of microglia from homeostatic to proliferative populations, while demonstrating a transition of granulocyte cells from G-MDSCs to mature neutrophils. By inducing the depletion of PMNs and MDSCs, an increase in the bacterial burden was observed [103].

Interestingly, G-MDSCs appear to be associated with implant infections but not with aseptic loosening. Therefore, their presence in peri-implant tissues could represent a distinctive mark of infection [104]. On the other hand, *S. aureus* bacteria in biofilms differentially modify their gene expression patterns, depending on the leukocyte subset encountered [86].

Overall, it has become apparent, from the latest research studies, that the anti-inflammatory environment that can develop in prosthetic joint infections caused by *S. aureus* is characterized by a high local recruitment of MSDSCs and an increased production of IL-10 [79]. In this connection, *S. aureus* has recently been found to impair neutrophil functions by enhancing the expression of the immune response gene 1 (*Irg1*), which encodes the enzyme implicated in the synthesis of a key anti-inflammatory metabolite, namely itaconate [104]. In host immune cells, the upregulation of the itaconate synthetic enzyme is associated with a mitochondrial oxidative stress, induced by staphylococcal glycolysis. Furthermore, it has been shown that high levels of itaconate are able to suppress the oxidative burst of neutrophils, to inhibit neutrophil degranulation, to promote capsular exopolysaccharide synthesis in bacteria, and, therefore, to lead to an abundant formation of bacterial biofilms [104,105,106,107]. Other interesting findings concern the activity of lactate derived from the metabolism of *S. aureus* biofilms. In murine MDSCs and macrophages, bacterial lactate was found to inhibit the enzyme histone deacetylase 11 (HDAC11). This inhibition would result in unchecked HDAC6 activity and increased histone 3 acetylation at the IL-10 promoter, leading to increased IL-10 expression [108]. These observations are consistent with the elevated amounts of lactate and IL-10 in the synovial fluid of patients with prosthetic joint infections [108].

**Figure 2:**
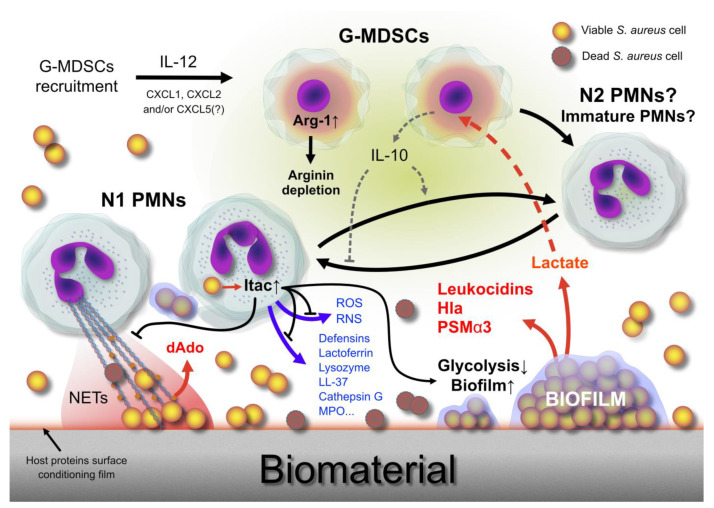
In recent years, there have been considerable advances in the knowledge of the interactions between pathogens and host cells at the infection sites. The role of G-MDSCs and of different subsets of leucocytes exhibiting distinct phenotypes (e.g., N1 and N2 PMNs) has attracted growing interest, and so has the ability of pathogens to hijack the host’s immune defenses. The latest discoveries have highlighted the critical importance of immunometabolism and of the metabolic interplay between pathogens, such as *S. aureus*, and the host immune cells. This illustration attempts to illustrate the intricate network of interactions between *S. aureus* and PMNs that take place at the tissue–implant interface. The emerging role in the immunometabolism of two metabolic products, respectively the lactate (produced by *S. aureus* biofilm) and the itaconate (produced by PMNs following the interaction with *S. aureus*), is featured. Legend: Arg-1, arginase-1; ROS, reactive oxygen species; RNS, reactive nitrogen species; Itac, itaconate; PSMα3, phenol soluble modulin alpha 3; dAdo, 2′-deoxyadenosine.

The mechanisms of leukocyte immunometabolism reprogramming and the hijacking of the host immune response by *S. aureus* and *Pseudomonas aeruginosa* have been recently reviewed by Souche et al. [106], in the context of cystic fibrosis. Figure 2 attempts to summarize the complex interactions taking place at the implant–tissue interface, some of which were only recently unveiled, between *S. aureus*, the host’s G-MDSCs, and neutrophils.

Besides secreting a plethora of molecules to evade the immune defenses, *S. aureus* can acquire mobile genetic elements when it is in the crowded community of a bacterial biofilm. To safeguard their survival and preserve their replication, staphylococci are also able to redirect host biological activities, such as fibrin formation or even NET formation (see below).

## 7. Biofilm Shielding and Frustrated Phagocytosis

Now, we briefly consider the role and significance of biomaterials in biofilm-associated infections and highlight how their presence impacts the neutrophil–biofilm interactions. It should be emphasized that biomaterial is the starting point of the infection as it constitutes the compliant substrate of proteins adsorption, bacterial adhesion, and biofilm formation [14]. Bacterial adhesion and biofilm production proceed in two stages. First, bacterial adhesins mediate the interaction between the bacterium and the extracellular matrix proteins that film the biomaterial surface. Hence, an adhesin-mediated anchoring allows bacteria to adhere to the surface of the biomaterial and to produce biofilm. The physical and chemical characteristics of the biomaterial influence the bacterial adhesion and biofilm formation. In this context, the development of anti-infective biomaterials and anti-adhesive surfaces is the main strategy to prevent biofilm formation, increasingly perfecting the biomaterial anti-biofilm properties together with its safety for eukaryotic cells [14,109,110,111,112,113].

In vivo, in the presence of an implant, the host immune response may prove inadequate to comprehensively clear infections caused by biofilm-forming bacteria. Thus, infections turn chronic or sub-chronic. The mechanisms that determine the bacterial resistance of biofilms to leukocytes were, at first, explained either by a lack of penetration (exclusion) of leukocytes into biofilms, or by a decreased ability of phagocytes to actively kill the biofilm-encased bacteria, a process termed “frustrated phagocytosis” [73,114]. Early studies on *S. aureus* biofilms found that human leukocytes effectively penetrate biofilm [115], but they exhibit impaired phagocytosis and a decreased bactericidal ability. Conversely, in other investigations, PMNs recognized biofilms and activated defense-associate reactions, including phagocytosis, degranulation, and DNA release, showing that biofilms are not inherently protected against the attack by phagocytic cells [116].

Hänsch et al. studied the behavior of polymorphonuclear neutrophils towards staphylococcal biofilms and demonstrated that neutrophils enter and destroy biofilms, but they also damage host tissues at the site of infection [117,118]. The authors suggested that both soft tissue damage and osteolysis are not a direct effect of infection but depend on the proinflammatory environment created by the immune cells.

At the site of infection, neutrophils enhance the production of reactive oxygen species, while the migration of neutrophils from blood vessels and their chemotaxis are significantly reduced. It can be assumed that neutrophils first move and migrate in response to infection, then they slow down the movement but exacerbate the expression of their powerful armamentarium of bactericidal, cytotoxic, and proteolytic factors, with which they also compromise the integrity of the surrounding tissues. The “stay and fire” attitude that neutrophils exhibit in implant-associated infections correlates with different factors. The first is the upregulation of surface receptors for bacterial recognition and killing, such as the high-affinity Fc-gamma 1 receptor (FcγRI, CD64) and the CD14 “lipopolysaccharide” receptor. A second is the downregulation of adhesion molecules, such as L-selectin (CD62L), essential for the adhesion of neutrophils to endothelial cells, as adhesion is the prelude to neutrophils’ diapedesis and migration. A third is the strong up-regulation of IL-8 and of the inflammatory proteins of monocytes (MIP-1α and MIP-2β) [14].

In in vitro studies, neutrophils surround biofilms and activate, but do not migrate freely and easily into the biofilms due to unfavorable circumstances, such as the reduction in chemotactic molecules and the encumbrance created by the “slimy” substance of the biofilm matrix. Even in vivo, neutrophils show reduced chemotaxis. They attempt to penetrate through the biofilms accumulated on the surfaces of biomaterials, but they encounter obstacles in reaching and engulfing the deeply biofilm-enclosed bacteria. Thus, they cannot perform a complete clearance of biofilm. The properties of the biofilm extracellular matrix motility seem to influence the anti-biofilm activity of neutrophils. Indeed, the latest findings on the structural composition of biofilm show distinct biofilm matrices in different strains belonging to the same bacterial species and even in the same bacterial strain under different environmental conditions [119]. Rybtke et al. found that the extracellular matrix polysaccharides in *P. aeruginosa* biofilm played a role in the response of PMNs toward biofilms. More precisely, *P. aeruginosa* mutants that were able to produce a subset of matrix exopolysaccharides elicited distinct PMN responses. The PMNs responded aggressively toward a biofilm matrix consisting of both Psi and alginate exopolysaccharides [120].

Differences have emerged in the phagocytosis of *S. aureus* and *S. epidermidis* biofilms by PMNs [114], due, presumably, to the properties of their biofilm matrix. *S. aureus* biofilms appear more susceptible to PMNs than *S. epidermidis* biofilms, in which PMNs appear less mobile [121]. Time-lapse microscopy showed that PMNs moved across the *S. aureus* biofilm and took up bacteria. In the *S. epidermidis* biofilm, PMNs, rather immobile, only phagocytosed bacteria in the immediate proximity [121].

Another facet of the complex interaction between neutrophils and biofilm is the influence exerted on this interaction by the level of biofilm maturation. This property seems to influence in vitro (and presumably in vivo) the extent of biofilm clearance.

Alhede et al. explored if the size of aggregates is critical for successful phagocytosis and studied how bacterial biofilms evade phagocytosis. They investigated the interaction between PMNs and *S. aureus*, *S. epidermidis*, *Escherichia coli*, and *P. aeruginosa* by means of confocal scanning laser microscopy. They found that the size of the aggregate significantly affected phagocytosis and that neutrophils had difficulties phagocytosing the larger aggregates. When aggregates become too large, circulating PMNs may not be able to phagocytose them quickly enough, which may lead to chronic infection [122].

In an in vivo model of implant-associated infection by *S. aureus*, biofilm formation started on the first day after implant surgery and then gradually increased, reaching the maximum around the fourteenth day. Then, biofilm growth declined, and the biofilm stabilized for the duration of the study (6 weeks) [123]. Therefore, the interval of time in which the bacteria are still in the planktonic phase and are therefore more vulnerable has a very short duration.

Immature biofilms are more susceptible to neutrophil phagocytosis than mature ones, although even mature biofilms appear rather vulnerable to PMN cells [116]. Neutrophils moved towards mature biofilms, and adhered and responded (for example, by releasing cytokines) to biofilms, even if under difficult conditions, such as those of physiological shear stress. Neutrophils also attempted to engulf the copious, well-anchored, and deeply rooted biofilms that grow on biomaterials, often unsuccessfully and with side effects.

## 8. Collateral Tissue Damages

Lack of tissue integration and prosthetic loosening are often adverse reactions associated with septic failure [124]. In the infection site, neutrophil chemotaxis and migration from the blood vessels decrease, while the production of reactive oxygen species is enhanced [125]. Particularly, a strong up-regulation of the migration inhibitory factor-related protein 14 (MRP-14), a cytokine able to induce the differentiation of monocytes to osteoclasts, has been reported [126].

Metal wear particles, even in non-toxic doses, can concur to stimulate the release of pro-inflammatory mediators, with consequent osteoclast proliferation and bone resorption. Through in vitro experiments, Dapunt et al. investigated the effect of metal wear particles on human neutrophils, monocytes, and osteoblasts: small amounts of bacterial components with metal wear particles enhanced the inflammatory response in immune cells and in osteoblasts [127]. Importantly, in implant infections, neutrophils massively infiltrated the infected site, with the number of neutrophils correlated with that of bone-resorbing osteoclasts. All the above observations are indirect signs of a correlation between the proinflammatory action of biofilm-challenging neutrophils and bone loss [122].

Neutrophils act as if they were always at war: numerous, enemy-encircling, armed, firing. In doing so, they also cause collateral damages. By combining bioluminescent and fluorescent optical imaging with X-ray and μCT imaging, the dynamic changes in bacterial burden, neutrophil recruitment, and bone damage have been documented in a mouse orthopedic implant infection model. There was evidence that the persistence of bacterial biofilms on the implanted materials caused inflammation, periprosthetic osteolysis, and osteomyelitis [128].

Interestingly, there are also silent implant infections. Indeed, instances have been reported of implants that were colonized by bacteria without producing symptoms or even revealing patent clinical signs of infection [129,130,131]. This occurrence may mean that certain biofilms remain indolently on implants over years, by establishing a sort of co-existence with immune cells. Supposing this is true, the fascinating question is the following: how is it possible? Up to now, this point remains not exhaustively addressed in literature and, thus, a presumed quiescence of dormant biofilms, or continuous, but not radical, biofilm cleansing by immune cells, could be the cause of those infections that remain paucisymptomatic/asymptomatic for a long time [130,132,133,134,135].

## 9. Neutrophil Extracellular Traps (NETs) and Counteractions by *S. aureus*

As aforementioned, NET formation is a tool used by PMNs to fight bacteria by trapping and confining them [40]. Many very recent papers have reported on NETs and biofilms, illustrating the mechanisms that pathogens (mainly staphylococci) have contrived to escape them [78,79,136,137].

Another interesting review recognizes NETs in animals and root extracellular traps (RETs) in plants as important players in immunity [138].

**Figure 3:**
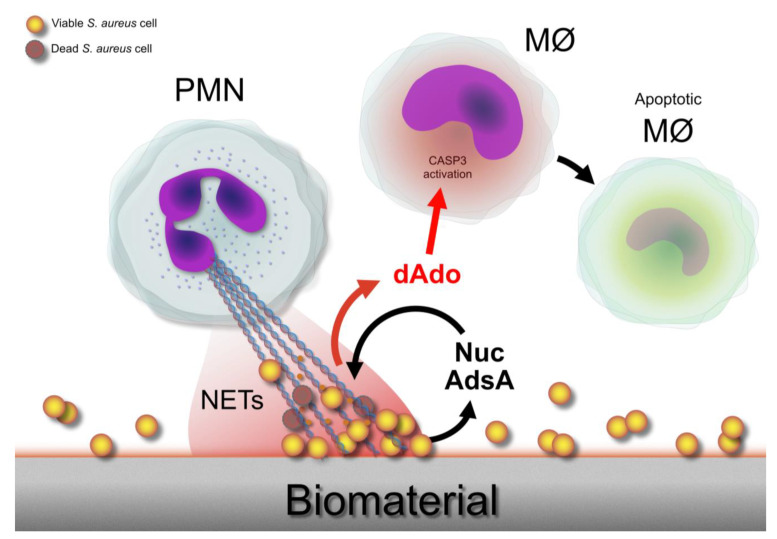
Following cellular activation, PMNs undergo NETosis and release their “DNA-based traps”, termed NETs, in the extracellular space. Largely consisting of DNA filaments and antimicrobial components such as PMNs, granules, and antimicrobial peptides, NETs tangle and snare bacterial cells. Nonetheless, for evolved pathogens such as *S. aureus*, the NETs’ bactericidal activity is limited. Indeed, *S. aureus* cells can disentangle and remove themselves NETs by expressing the enzyme nuclease (Nuc), degrading NETs DNA filaments. Moreover, in the process of DNA degradation, the leukocidal proapoptotic substance 2′-deoxyadenosine (dAdo) is produced by the action of the enzyme adenosine synthase A (AdsA), thus backfiring. dAdo-mediated killing has been found to occur in macrophages (MØs) through the activation of the caspase-3 (CASP3) pathway.

The complex interplay between staphylococcal biofilms and NETs emerges from the finding that molecules involved in biofilm dispersal are capable of degrading NETs [139,140]. The best strategy to evade the immobilizing traps of neutrophils is, indubitably, to destroy them. Since the main component of NETs is DNA, DNases are enzymes crucial for the virulence and survival of several bacterial species, including S. aureus. DNases can efficiently degrade NETs. *S. aureus* is able to enzymatically convert the DNA derived from NETs to 2′-deoxyadenosine, a molecule able to trigger apoptosis in immune cells [141]. Thus, unfortunately for neutrophils, *S. aureus* “returns fire” when they cast their nets (Figure 3).

A recent study showed that cancer cells can induce neutrophils to undergo NETosis, to shield themselves from attack by immune cells [142].

Bhattacharya et al. described key aspects of the interaction between primary human neutrophils and *S. aureus* biofilms and provided insight into how *S. aureus* evades the neutrophil response to cause persistent infections. They attributed the persistence of biofilm bacteria trapped in NETs to the leukocidin LukAB, which promotes *S. aureus* survival during phagocytosis, and to Nuc, which degrades NET DNA [136].

## 10. Biofilm Matrices

In the struggle of neutrophils against staphylococci embedded in biofilms, the outcome of the battle also depends on the composition of the biofilm matrix and on its modulation and maturation. Proportions, quantity, and quality of the main macromolecular components (i.e., polysaccharides, proteins, eDNA, and teichoic acid) of biofilm matrices vary greatly among different environmental contexts, biofilm growth phases, bacteria species, and even strains of the same species.

Different compositions of biofilm matrices result in different types of molecular interactions within the peculiar biofilm matrix and have different repercussions on the interactions with neutrophils [120].

## 11. Pattern Recognition Receptors and Pathogen-Associated Molecular Patterns

Cells of the innate immune system recognize highly conserved pathogen-associated molecular patterns (PAMPs) that are expressed by many microorganisms, staphylococci included [137]. Receptors of the innate immune system, called pattern recognition receptors (PRRs), recognize these conserved bacterial motifs. Toll-like receptors (TLRs) are a PRR class expressed by cells of the innate immune system. TLRs mediate cellular activation in response to PAMPs [138]. The role for TLRs in mediating innate immune recognition of staphylococcal species during planktonic growth has been well-characterized [139].

Biofilm-encased bacteria, wrapped up as they are in a complex three-dimensional structure with few bacteria exposed at the outer surface, are not open to detection by Toll-like receptors (TLRs), which are expressed on the surface of phagocytes. Thurlow et al. showed that the *S. aureus* biofilm is able to circumvent, at least partly, the recognition of TLR2 and TLR9 [114]. The underlying mechanism is unknown, but it could be ascribed to the interference of biofilm matrix components with the optimal ligand–TLR engagement. Thus, the exopolysaccharide of the *S. epidermidis* biofilm matrix could hinder the interaction with neutrophil receptors. This is not surprising for a bacterium that relies on its polysaccharide biofilm to survive, that possesses an entire locus to ensure polysaccharide production [140], and that is even able to turn on/off biofilm production to adapt to hostile environmental conditions [141,142]. Similarly, the fibrin that *S. aureus* incorporates into its biofilm, by using coagulase enzyme to convert fibrinogen to fibrin, could protect this coagulase-positive bacterium by the immune recognition [143].

Neutrophils can produce many cytokines and chemokines, which modulate the inflammatory response as well as the immune response [144,145]. Moreover, iNOS (inducible nitric oxide synthase) levels appeared significantly reduced in biofilm infections, in which, instead, arginase-1 expression dominated. Thus, in biofilm infection, the skewing from iNOS to an arginase-1 response could be another explanation of why biofilm infections can persist in an immunocompetent host [114].

Multiple studies showed many evasion strategies, although these are often referring to planktonic bacteria. Nevertheless, understanding them can also help to enlighten us about neutrophil–biofilm interactions, as it is likely that some evasion mechanisms occur in biofilms as well, or could concur to biofilm formation by hiding, from the sight of neutrophils, those planktonic bacteria that subsequently will organize themselves into a biofilm community.

Nevertheless, it can be assumed that, in most cases of host tissue bacterial contamination in immunocompetent hosts, neutrophils intervene at the right time, possibly when bacteria are still in their more vulnerable planktonic stage or are only just beginning to form biofilm.

## 12. Neutrophils as Therapeutic Targets: Can Immune Suppression and Immune Resolution Be Helpful in Implant Infections?

As previously described, neutrophils sense signals from the infected and/or damaged pro-inflammatory milieu and, consequently, orchestrate immune responses. Neutrophil-driven chronic inflammation is a recognized pathogenetic mechanism for various chronic diseases [146], among which osteoarthritis, rheumatoid arthritis and other autoimmune diseases, atherosclerosis, diabesity, Alzheimer’s disease, cancer, and, finally, peri-prosthetic infections are included. This finding seems to suggest that chronic diseases suppress the neutrophils’ activities. But we cannot neglect to consider that all immune manipulations will each provide either beneficial effects or detrimental effects, depending on a multiplicity of factors. In implant infections, the major issue concerning neutrophils is not merely that of extinguishing the fire of their persistent pathological response, but also, ideally, that of preserving (or, even better, strengthening) the neutrophils’ ability to fight infection. Therefore, the crucial role of neutrophils in antibacterial host defense prejudges the usefulness of curbing their responses.

The deficit of immune resolution (i.e., the phase that naturally concludes a successful acute inflammatory response) is increasingly recognized as an important trigger for chronic destructive inflammation. Exploiting pro-resolving mediators or using pro-resolving-based drugs are new emerging strategies to activate the immune resolution process. Pro-resolving mediators (mapped in [147]) include the lipids resolvins, lipoxins, protectins, and maresins; the proteins annexins and galectins; and the gaseous molecules hydrogen sulfite and carbon monoxide. Pro-resolving-based drugs are drugs that mimic or potentiate the effects of natural pro-resolving mediators (reviewed in [148]). Inducing or favoring immune resolution could be a more cautious alternative strategy to the immune suppressive one. However, in implant-associated infections, the perpetuation of inflammation, with the consequent lack of immune resolution, is due to the presence of a biofilm that frustrates phagocytosis rather than an inherent dysfunction of neutrophils. Therefore, evoking immune resolution does not seem to be a convincing and exhaustive approach either.

Further elements of ambiguity, and therefore reasons for caution, derive from the growing evidence on the ability of biofilms to divert neutrophil defenses [149] or to curb their activity [104,105,106,107,108].

## 13. Anti-Infective Biomaterials

Referring to what has emerged here on the interactions of neutrophils with biofilms in the periprosthetic environment, the most concrete possibility of helping neutrophils remains that of using anti-infective biomaterials. These include repellent biomaterials able to prevent contact with bacteria; biomaterials that kill contacting bacteria (the so-called contact killing); and biomaterials that release antibacterial agents to target bacteria at a distance [150,151]. Thus, neutrophils can face bacteria on a more level playing field, i.e., before bacteria have had a chance to use their adhesion and evasion mechanisms and, more importantly, before they have settled down and organized into thick and solid barricades.

Furthermore, loading new alternative-to-antibiotic antimicrobial agents onto biomaterials appears to be suitable for overcoming bacterial resistance to antibiotics and promoting periprosthetic tissue regeneration, which is particularly desirable for the osseointegration of orthopedic implants. In this connection, new bioinspired molecules, like the ones released by neutrophils (such as antimicrobial peptides), and the plant-derived ones (carvacrol, curcumin, etc.) appear to be promising tools for designing anti-infective pro-regenerative biomaterials [152,153,154,155].

## 14. Conclusions

In most cases, neutrophils intervene when bacteria are in their planktonic phase, which is a more vulnerable condition than when they are within a biofilm. Surface-modified biomaterials, functionalized with anti-infective molecules to repel or kill bacteria, can help neutrophils by preventing bacteria from colonizing and forming biofilms.

But even if thwarted by implant biofilms and other bacterial countermeasures, neutrophils are not always the losers.

Although pathogens such as *S. aureus* possess a multiplicity of virulence factors, by which they can hide in plain sight [156], hijack neutrophils [149], degrade their traps [157], laugh in their face [158] and, finally, kill them, for their part neutrophils are not discouraged and, by activating themselves, fight against biofilms.

When they are not capable of phagocytosing biofilms via acute inflammation, a chronic inflammatory process takes over, with its sluggish course. Frustrated phagocytosis leads to the functional exhaustion of immune cells and subsequent tissue damage. Hence, biofilm persists, bone degradation continues, and the implant heads towards loosening and failure [159].

## Data Availability

Data are contained within the article.
